# Supplementary material for: Comparative Root Transcriptomics Provide Insights into Drought Adaptation Strategies in Chickpea (Cicer arietinum L.)
Source: Int J Mol Sci. 2020 Mar 5;21(5):1781. doi: 10.3390/ijms21051781 (PMC7084756; doi:10.3390/ijms21051781)
Supplement: Supplementary file 1 [file ijms-21-01781-s001.zip › ijms-711422 suppl for final/suppl figure S1 and S2.pdf]

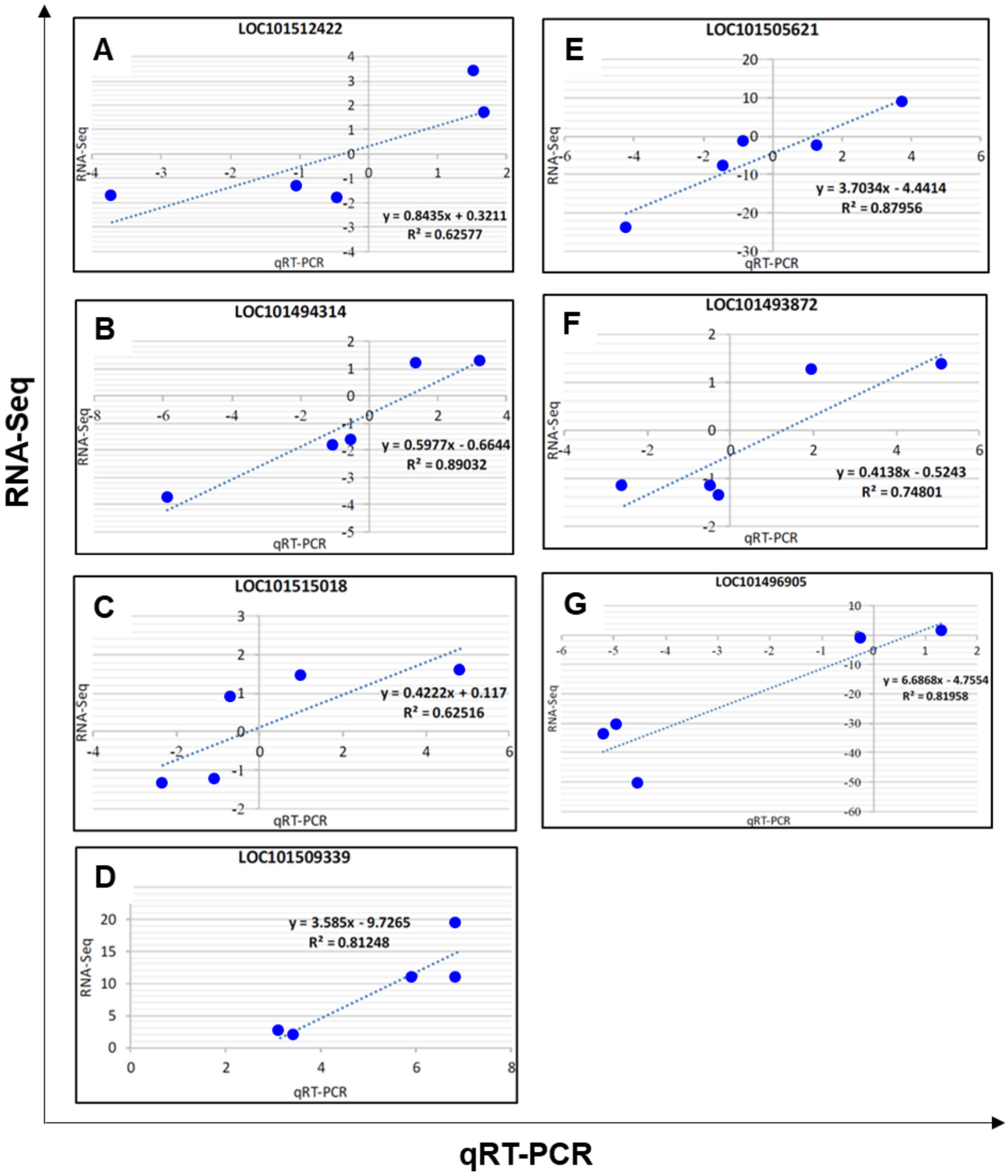

Supplementary Figure S1: qRT-PCR validation of DEGs during three developmental stages VS, RTS, RS. The correlation between fold change obtained by using qRT-PCR (x-axis) and RNA-Seq (y-axis).

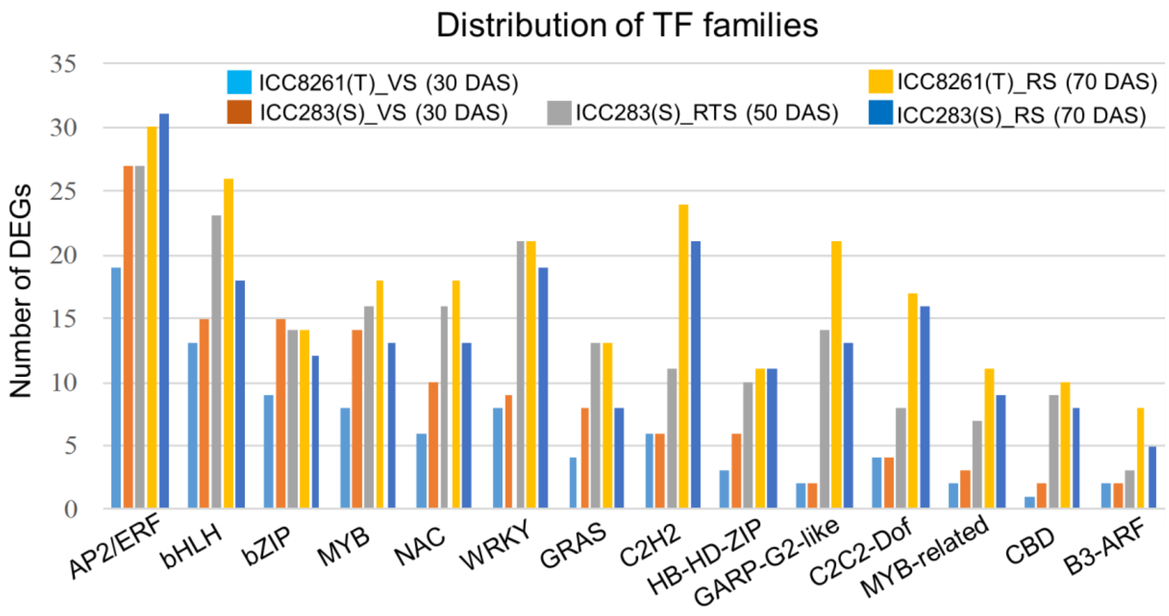

Supplementary Figure S2: Top TF families represented in DEGs across vegetative stage (VS), reproductive transition stage (RTS) and reproductive stage (RS) in tolerant (T, ICC8261) and sensitive (S, ICC283) genotypes.
